# Less pain does equal better quality of life following strontium-89 therapy for metastatic prostate cancer

**DOI:** 10.1054/bjoc.2000.1610

**Published:** 2001-02

**Authors:** S L Turner, S Gruenewald, N Spry, V Gebski, on behalf of the Metastron Users Group

**Affiliations:** Department of Radiation Oncology, Westmead Hospital, NSW, 2145, Australia; Department of Nuclear Medicine, Westmead Hospital, NSW, 2145, Australia; Department of Radiation Oncology, Charles Gairdner Hospital, Perth, Western Australia 6000; NHMRC Clinical Trials Centre, Sydney University, NSW, 2006, Australia

**Keywords:** quality of life, strontium-89, bone pain, prostate cancer

## Abstract

93 patients with hormone refractory metastatic prostate cancer were entered on a prospective study to measure reduction in pain and changes in quality of life (QoL) after the administration of 150 MegaBequerel (MBq) Strontium-89 (Sr-89). QoL was assessed using a validated instrument, the Functional Living Index – Cancer (FLIC) questionnaire. Pain response was measured using the Radiation Therapy Oncology Group scoring system. Overall there was limited QoL improvement over 3 months following Sr-89. However, in the 53 patients (63%) achieving pain responses, QoL did significantly improve within 6 weeks of receiving Sr-89 compared to patients with stable or worsening bone pain, and this was independent of other parameters that might influence QoL outcomes, such as performance status, baseline PSA and extent of skeletal disease (*P* = 0.004). PSA ‘response’ occurred in 30 patients (37%) over 4 months after Sr-89. This did not appear to correlate with clinical improvement. This study supports the presumption that improvement in pain following Sr-89 is accompanied by better QoL. The lack of correlation of PSA response and clinical parameters indicates that in the palliative setting, PSA may not provide a useful surrogate for treatment outcome. © 2001 Cancer Research Campaign http://www.bjcancer.com


British Journal of Cancer (2001) 84(3), 297–302
© 2001 Cancer Research Campaign
doi: 10.1054/ bjoc.2000.1610, available online at http://www.idealibrary.com on IDEAL®

### Less pain does equal better quality of life following strontium-89 therapy for metastatic prostate cancer

**SL Turner[1], S Gruenewald[2], N Spry[3] and V Gebski[4] on behalf of the Metastron Users Group**

[1]Department of Radiation Oncology, Westmead Hospital, NSW, 2145, Australia; [2]Department of Nuclear Medicine, Westmead Hospital, NSW, 2145, Australia; [3]Department of Radiation Oncology, Charles Gairdner Hospital, Perth, Western Australia 6000; [4]NHMRC Clinical Trials Centre, Sydney University, NSW, 2006, Australia

**Summary** 93 patients with hormone refractory metastatic prostate cancer were entered on a prospective study to measure reduction in pain and changes in quality of life (QoL) after the administration of 150 MegaBequerel (MBq) Strontium-89 (Sr-89). QoL was assessed using a validated instrument, the Functional Living Index – Cancer (FLIC) questionnaire. Pain response was measured using the Radiation Therapy Oncology Group scoring system. Overall there was limited QoL improvement over 3 months following Sr-89. However, in the 53 patients (63%) achieving pain responses, QoL did significantly improve within 6 weeks of receiving Sr-89 compared to patients with stable or worsening bone pain, and this was independent of other parameters that might influence QoL outcomes, such as performance status, baseline PSA and extent of skeletal disease ($P = 0.004$). PSA ‘response’ occurred in 30 patients (37%) over 4 months after Sr-89. This did not appear to correlate with clinical improvement. This study supports the presumption that improvement in pain following Sr-89 is accompanied by better QoL. The lack of correlation of PSA response and clinical parameters indicates that in the palliative setting, PSA may not provide a useful surrogate for treatment outcome. © 2001 Cancer Research Campaign http://www.bjcancer.com

**Keywords**: quality of life; strontium-89; bone pain; prostate cancer

Several phase III studies (Quilty et al, 1982; Lewington et al, 1991; Porter et al, 1993; McEwan et al, 1994; Malmberg et al, 1997) now support the usefulness and cost effectiveness of Sr-89 either as an alternative or an adjunct to external beam radiotherapy (EBRT) in men with bone pain due to metastatic prostate cancer. Primary study end points have included pain response presented in terms of reduction in severity and/or frequency of pain and/or analgesic use, delayed onset of new painful sites and/or decrease in EBRT requirement over the patient’s remaining lifetime.

It is recognized that the effects of palliative treatment extend beyond simply reducing the severity of a reference symptom, such as pain. Health-related QoL describes a multidimensional view of a patient’s psychological and social, as well as physical wellbeing. Despite the frequent citing of the importance of overall QoL in men with metastatic prostate cancer and the potential impact that Sr-89 might have on QoL, there has been little attention paid to formal evaluation of QoL as a primary end-point in the study of Sr-89 to date.

The Trans Canada Study Group (Porter et al, 1993) undertook a limited QoL evaluation and found that in the areas of physical mobility and pain control, Sr-89 produced a significant beneficial effect for men with metastatic prostate cancer. This assessment, as the authors acknowledge, was not ideal in that a standardized instrument was not used and conclusions were based on data pooled from a number of non-validated instruments including linear analogue self assessment scales, questionnaires and patient diaries.

*Received 2 May 2000*
*Revised 16 October 2000*
*Accepted 27 October 2000*

*Correspondence to:* SL Turner

Following the establishment of a Government reimbursement in early 1995, Sr-89 therapy for the relief of bone pain due to prostate cancer became widely available in Australia. A group of interested Radiation Oncologists (the Australian Metastron User’s Group) took this opportunity to initiate a prospective study to (1) confirm that treatment efficacy and toxicity were consistent with the world literature and to (2) simultaneously assess the nature and time course of any possible QoL benefits using a validated instrument. Secondary end-points included toxicity, (specifically bone marrow depression, pain ‘flare’ and/or other unexpected morbidity), and PSA ‘response’ following Sr-89.

### MATERIALS AND METHODS

This was a prospective multicentre study involving 13 oncology centres in 5 states and territories of Australia. Accrual took place over a two-year period from mid 1995 to mid 1997. There was a minimum 8-month interval from the date of Sr-89 injection in the last study patient to analysis, with a median follow up overall of 6.8 months (censoring patients at death).

Eligible patients were those who had histologically proven prostate cancer with bone scan evidence of metastatic disease and who had progressed either clinically, biochemically or both following ‘standard’ first-line hormone therapy (with or without further hormonal manoeuvres), and who had one or more sites of bony pain. 7 patients had previously been treated with various chemotherapy agents as well.

The protocol deliberately did not specify the exact indication for the use of Sr-89 so as to reflect general clinical practice. However, Sr-89 was not approved at that time for use as an adjunct to external beam radiotherapy or for men with asymptomatic bone metastases only. Patients were required to have platelet levels $\geq 100 \times 10^9$ $l^{-1}$ and white cell counts $\geq 3.5 \times 10^9$ $l^{-1}$ prior to delivery of Sr-89. Ethics committee approval was granted by involved institutions and patients were required to provide written consent.

At registration, baseline QoL and pain evaluations were undertaken. QoL was measured using the University of Manitoba, Winnipeg, Functional Living Index – Cancer Instrument (FLIC), Schipper, 1987. This is a well-validated, easily applied self-assessment questionnaire incorporating multiple facets of psychosocial state and sociability as well as parameters of physical well being (Appendix B). Furthermore, this instrument was of particular interest in that the validation studies of FLIC report lack of correlation between traditional assessments of response, for example improvement in pain, and other areas impacting on day-to-day function such as mood and relationships with others.

Pain was assessed jointly by physician and patient according to the Radiation Therapy Oncology Group (RTOG) criteria (Appendix A), (Tong et al, 1982). Analgesic requirements prior to and after delivery of Sr-89 were recorded and used in conjunction with pain scores to establish pain response over the time intervals examined. Patients who had increase in analgesic requirements were not scored as having pain improvement, regardless of RTOG pain score status.

Other baseline parameters including performance status, PSA at recruitment, time since diagnosis of metastases, and extent of skeletal disease (greater or less than 20 bone scan ‘hot spots’) were recorded to allow correlation of these measures of disease status at time of Sr-89 therapy with QoL responses. It was hypothesized that patients who were earlier in the course of their metastatic disease and/or with better performance status might benefit more from Sr-89 than those who were not. In addition, it was postulated that biochemical (PSA) changes, if still a useful surrogate for disease activity in advanced disease, might parallel pain response.

Patients received a single 150 MBq intravenous dose of Sr-89. Follow up assessments of pain and QoL were conducted at between two and three weeks following the injection and then monthly if possible, until death. Toxicity, using WHO/EORTC criteria for bone marrow suppression (Miller et al, 1981) and PSA data were also recorded on these occasions. Where monthly visits to the clinic were deemed inappropriate either on geographic or medical grounds, then some information was collected by mailed FLIC questionnaires and telephone conversations with patients or carers.

One patient was lost to follow up at the time of analysis. 8 patients had no post-treatment pain scores recorded (4) or response data was only available beyond 3 months after Sr-89 (4). 11 patients had no follow-up QoL data collected. This left 85 patients evaluable for pain response and 82 patients evaluable for QoL analysis.

The time intervals chosen for examination of QoL and pain response were 0 to 6 weeks from Sr-89 (as any beneficial effect of treatment would be expected to be seen well within this period), 6 weeks to 3 months (as most investigators report this as a median period for maintaining pain response to radionuclide treatment) and from 3 months to the time of death. The number of surviving patients in the last time interval was too small to meaningfully report results.

Time to event (death) was measured from the day of the Sr-89 injection, with survival curves calculated using the method of Kaplan and Meier (1958). For the follow up periods of 0 to 6 weeks and 6 weeks to 3 months, patients with multiple QoL measures had their QoL scores averaged within individuals. Factors considered as potentially influencing QoL changes included ECOG performance status, number of hot spots and PSA at baseline, as well as time from diagnosis of bone metastases and pain response to therapy. Univariate and multivariate comparisons were performed on these parameters using multiple linear regression and where appropriate multiple logistic regression analysis.

### RESULTS

### Pain response

Pain response to Sr-89 was categorized as complete, moderate or minimal according to the RTOG pain scoring criteria (Appendix A). Overall pain response (any reduction in RTOG pain score) over the first 6 week and 6 week to 3 month periods taken together was 53 (62.4%) of 85 evaluable men. The degree of response is recorded in Table 1. Complete relief of pain (no analgesics required) was reported by 15 men (17.6%).

### Quality of life analysis

The FLIC index was observed to increase by a median of 2.3 points in 61 evaluable patients over the first time period and 12.8 points from baseline in 55 evaluable patients in the second time interval (6 weeks to 3 months). Examination of the possible influence of known prognostic factors is presented in the forest plots in Figure 1. None of these ‘early metastatic disease’ parameters nor performance status were found to statistically significantly relate to better QoL gain.

Patients achieving any pain response (minimal, moderate or complete) were statistically significantly more likely to have improvement in QoL on linear regression analysis ($P$ = 0.013). This relationship was sustained in a multivariate analysis adjusting for factors that might confound changes in QoL index such as measures of more advanced disease, as described, and poor performance status ($P$ = 0.004).

### Toxicity

In accordance with other investigators, pain ‘flare’ was defined as a temporary increase in pain following injection of radionuclide occurring between 0 and 14 days after the injection and lasting for between 1 and 14 days. 35 out of 90 evaluable patients (39%) had a flare, the majority occurring between 1 and 3 days following Sr-89 and lasting for 2 to 5 days.

10 patients experienced bone marrow depression that presented a clinical problem. One of these patients had oesophageal ulceration which bled 3 weeks after Sr-89. 3 patients became tranfusion-dependent and 7 others required at least one blood transfusion following Sr-89. Despite protocol stipulations and recommended

**Table 1** Pain response over three months following Sr-89

| Pain response | Number of patients | % |
|---|---|---|
| Complete | 15 | 17.7 |
| Moderate | 20 | 23.5 |
| Minimal | 18 | 21.2 |
| Stable/worsening pain | 32 | 37.7 |

Denominator varies according to slightly different numbers of patients evaluable within 6 week and 6 week to 3 month intervals.

 © 2001 Cancer Research Campaign

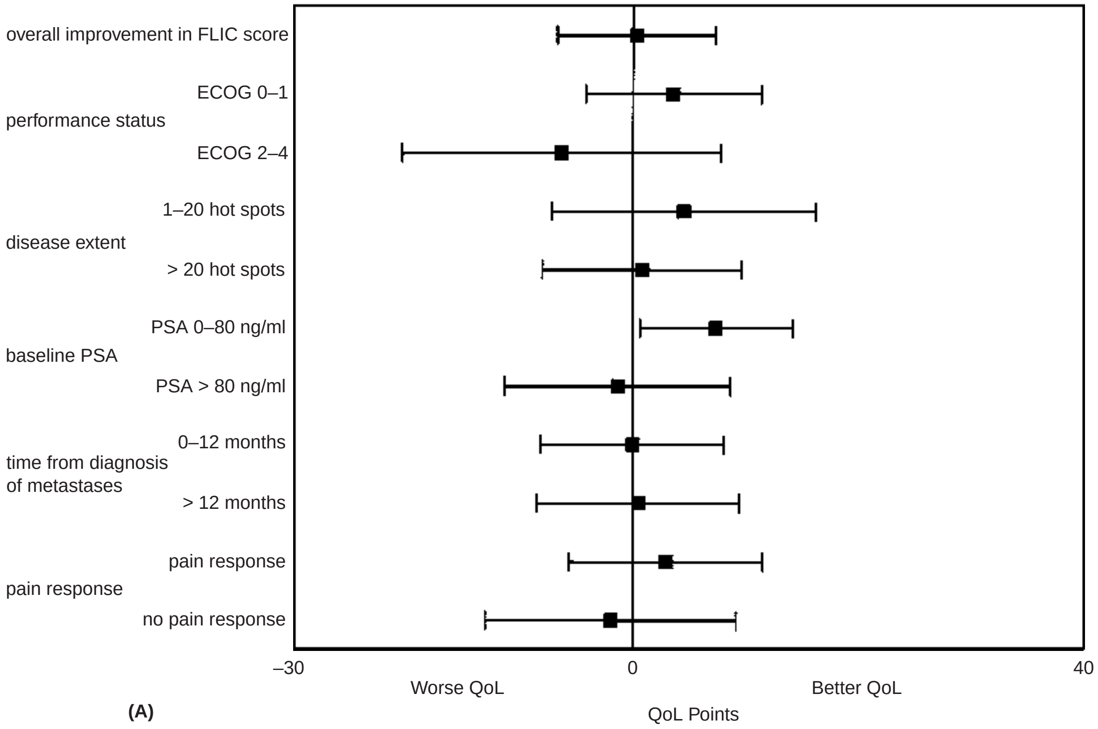

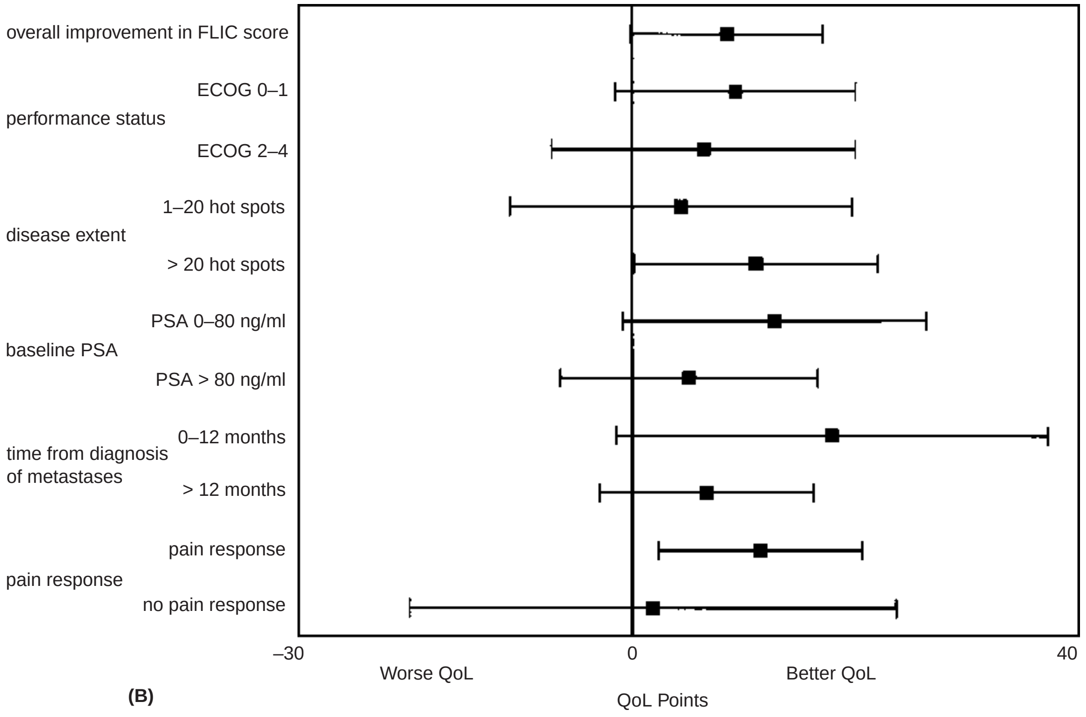

**Figure 1A** Changes in Quality of Life (FLIC) over the first six weeks (average in maximum difference from baseline with 95% confidence intervals). **B** Changes in Quality of Life (FLIC) between six weeks and three months (average in maximum difference from baseline with 95% confidence intervals).

guidelines for the use of this agent, only 49 patients (53%) had sequential white cell and platelet counts recorded following Sr-89. 6 of these (12% of evaluable patients) had post-treatment white cell counts of grade 2 or worse on at least one occasion. 11 patients (22% of evaluable patients) developed grade 2 or worse thrombocytopenia.

12 patients (13%) reported nausea and/or vomiting (10) or diarrhoea (2) which was felt by the treating physician and/or patient to possibly be attributable to Sr-89. Gastrointestinal disturbance occurred between 0 days and 2 weeks after injection of Sr-89.

© 2001 Cancer Research Campaign

Other adverse events included 7 cases of pathological fracture during the course of the patients' remaining lifetime. 10 patients developed malignant spinal cord compression (MSCC) at some time following injection of Sr-89. In none of these patients was MSCC known to be imminent at the time of Sr-89 – a contra-indication to the use of bone-seeking radiopharmaceutical. In 6 of these men, MSCC occurred within three weeks of the injection. One patient developed an attack of gout 7 days following Sr-89.

### PSA response

PSA 'responders' were defined according to the Trans Canada Study (Porter and McEvan, 1993) as being those in whom the PSA reduced at least 50% from the pre-treatment level. Depending on the time point examined, between 32 and 37% of evaluable men had PSA responses, thus defined, over 4 months following Sr-89, beyond which numbers of patients became too small to meaningfully analyse. PSA response seen in this study is presented in Figure 2 and is compared with the analogous Trans Canada results of both the active (400MBq Sr-89) and placebo arms. Numbers shown indicate the number of patients evaluable for assessment of PSA response at each time point.

Correlation of PSA responders and pain responders (of any degree), using a Chi-squared analysis did not find a statistically significant relationship between these two patient groups despite an initial trend (*P* values for 0 to 6 week and 6 week to 3 month intervals, 0.06 and 0.4 respectively).

There was no overall survival difference seen between men who had and those who did not have a PSA response.

### DISCUSSION

The response rates to Sr-89 therapy of 62.5% (including 17.5% complete responses) in this group of patients were typical of those reported in phase III (Lewington et al, 1991; Porter et al, 1993a; Quilty et al, 1994) and large phase II studies (Robinson et al, 1989; Laing et al, 1991) in the literature. This is despite the relative 'late' use of this agent in this study as judged by the high median baseline PSA (96 ng ml$^{-1}$), high proportion of patients with extensive skeletal disease (63% of 92 evaluable patients had more than 20 bone scan hot spots).

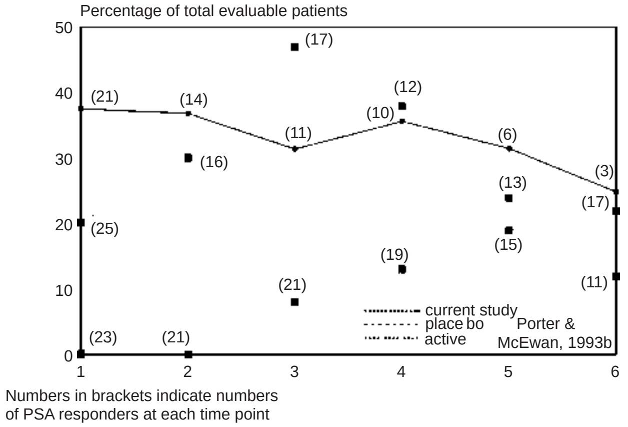

**Figure 2** Percentage of PSA responders over time following Strontium-89 (PSA response defined as at least 50% reduction in PSA from baseline – numbers in brackets indicate numbers of PSA responders at each time point)

Examination of the influence of prognostic factors on QoL score improvement (Figure 1) reveals some interesting trends. Whilst the confidence intervals are wide, the plots reveal that good prognosis patients (better ECOG, low baseline PSA and low bone scan load) tend to demonstrate larger increase in QoL score. This observation is in keeping with Laing et al (1991) who reported that a better analgesic effect was achieved with the administration of Sr-89 whilst skeletal disease load was relatively low. The trend for preferential benefit for good prognostic factors continued into the second period.

Although clinical experience and common sense suggest that QoL should improve in patients having reduction in pain, physical measures of response to therapies do not necessarily reflect their overall benefit to the patient. Demonstrating that QoL improvement really does accompany bone pain reduction following Sr-89 using objective testing is, therefore, important. In this study there was strong correlation with improved QoL for the first 6 weeks following Sr-89 therapy for patients in whom pain improved. This relationship remained highly statistically significant controlling for the above-mentioned indicators of more advanced metastatic disease and performance status.

The lack of demonstrable correlation between patients achieving pain responses and those in whom PSA reduced to 50% of its baseline level raises some points for contemplation. The exact mechanism of action of Sr-89 remains unknown but the results of this study would infer that the analgesic effect may be achieved through action on reactive bone cells around metastases or through effects mediated by other biological factors, rather than through direct cancer cell kill. Further, these findings suggest that either in this 'late' metastatic setting, PSA response is not a good surrogate of overall disease activity, or rather (and more likely), Sr-89 has a role in reduction of bone pain only without a substantial influence on the disease process as a whole.

On the other hand, the significant numbers of PSA 'responses' seen in this study, and in the Trans Canada Group report using Sr-89 at a higher dose, suggest that there is some cytotoxic or 'switching off' effect of Sr-89 on malignant prostate cells. This gives some support to the concept of a potential role for the earlier application of this radionuclide in the situation of subclinical or very early symptomatic metastatic disease, which was recently under investigation in Europe.

The toxicities recorded in this study point to the importance of pre-and post-treatment monitoring for patients receiving Sr-89, particularly in regard to bone marrow and neurological status.

In summary, this study suggests that we do improve metastatic prostate cancer patients' overall QoL with the use of Sr-89 when analgesic effect from this agent is achieved. As the current literature points to the advantage of early and adjunctive use of this agent in men with bone pain from prostate cancer metastases, it is beholden on clinicians treating these men to consider this therapy at the appropriate point in the time-course of their disease.

### ACKNOWLEDGEMENTS

Thanks to K Adams, P Apostolopolous and J Hallett for their assistance with data management and to M Skilleter, M Hughes and S Falvey for manuscript preparation. Thanks also to all participating clinicians, especially to Dr M Berry, Dr D Roos and Dr M Izard for their valued contributions.

 *© 2001 Cancer Research Campaign*

**REFERENCES**

Kaplan EL and Meier P (1958) Non parametric estimation from incomplete observations. *J Am Stat Assoc* **53**: 457–481

Laing AH, Ackery DM, Bayly RJ, Buchanan RB, Lewington VJ, McEwan JB, MacLeod PM and Zivanovic MA (1991) Strontium-89 chloride for pain palliation in prostatic skeletal malignancy. *Brit J Radio* **64**: 816–822

Lewington VJ, McEwan AJ, Ackery DM, Bayly RJ, Keeling DH, MacLeod PM, Porter AT and Zivanovic MA (1991) A prospective, randomised double-blind crossover study to examine the efficacy of strontium-89 in pain palliation in patients with advanced prostate cancer metastatic to bone. *Euro J Cancer* **27** (8): 954–958

Malmberg I, Persson U, Ask A, Tennvall J and Abrahamsson PA (1997). Painful bone metastases in hormone-refractory prostate cancer: Economic costs of Strontium-89 and/or external radiotherapy. *Urology* **50**(5): 747–753

McEwan AJB, Amyotte GA, McGowan DG, MacGillivray JA and Porter AT (1994) A retrospective analysis of the cost effectiveness of treatment with Metastron® (89 Sr-chloride) in patients with prostate cancer metastatic to bone. *Nuclear Medicine Communications* **15**: 499–504

Miller AB, Hoogstraten B, Staquet M and Winkler A (1981) Reporting results of cancer treatment. *Cancer* **47**(1): 207–214

Porter AT and McEwan AJB (1993) Strontium-89 as an adjuvant to external beam radiation improves pain relief and delays disease progression in advanced prostate cancer: Results of a randomised controlled trial. *Seminars in Oncology* **20**(3) Suppl 2: 38–43

Porter AT, McEwan AJB, Powe JE, Reid R, McGowan DG, Lukka H, Sathyanarayana JR, Yakemchuk VN, Thomas GM, Erlich LE, Crook J, Gulenchyn KY, Hong KE, Wesolowski C and Yardley J (1993) Results of a randomised phase-III trial to evaluate the efficacy of Strontium-89 adjuvant to local field external beam irradiation in the management of endocrine resistant metastatic prostate cancer. *International Journal of Radiation Oncology Biology Phys* **25**: 805–813

Quilty PM, Kirk D, Bolger JJ, Dearnaley DP, Lewington VJ, Mason MD, Reed NSE, Russell JM and Yardley J (1994) A comparison of the palliative effects of Strontium-89 and external beam radiotherapy in metastatic prostate cancer. *Radiotherapy and Oncology* **31**: 33–40

Robinson RG, Blake GM, Preston DF, McEwan AJ, Spicer JA, Martin NL, Wegst AV and Ackery DM (1989) Strontium-89: Treatment results and kinetics in patients with painful metastatic prostate and breast cancer in bone. *Radiographics* **9**(2): 271–281

Schipper H, Clinch J, McMurray A and Levitt M (1984) Measuring the Quality of life of cancer patients: the functional living index – cancer: development and validation. *Journal of Clinical Oncology* **2**(5): 472–483

Tong D, Gillick L and Hendrickson R (1982) The palliation of symptomatic osseous metastases. Final results of the study of the Radiation Therapy Oncology Group. *Cancer* **50**: 893–899

### APPENDIX A: RTOG PAIN SCORING SYSTEM

Pain score = product of severity and frequency.

**Pain severity**

0 – no pain

1 – mild pain

2 – moderate pain

3 – severe pain

**Pain frequency**
0 – no pain
1 – occasional (<once/day)
2 – intermittent (at least daily)
3 – constant (most or all of the time)

**Pain responses**
Minimal relief[a] – pain score dropping below initial score
Partial relief[a] – pain score dropping to ≤4
Complete relief – pain score dropping to 0 (no analgesics)

[a]Without significant increase in dose or type of analgesic.

### APPENDIX B: FLIC QUALITY OF LIFE QUESTIONNAIRE

**1. Most people experience some feelings of depression at times. Rate how often you get these feelings.**
**1 2 3 4 5 6 7 8 9 10**
Never … Continually

**2. How well are you coping with your everyday stress?**
**1 2 3 4 5 6 7 8 9 10**
Not Well … Very Well

**3. How much time do you spend thinking about your illness?**
**1 2 3 4 5 6 7 8 9 10**
Constantly … Never

**4. Rate your ability to maintain your usual recreation or leisure activities.**
**1 2 3 4 5 6 7 8 9 10**
Able … Unable

**5. Has nausea affected your daily functioning?**
**1 2 3 4 5 6 7 8 9 10**
Not At All … A Great Deal

**6. How well do you feel today?**
**1 2 3 4 5 6 7 8 9 10**
Extremely Poor … Extremely Well

© 2001 Cancer Research Campaign

**7. Do you feel well enough to make a meal or do minor household repairs today?**
**1 2 3 4 5 6 7 8 9 10**
Very Able — Not Able

**8. Rate the degree to which your cancer has imposed a hardship on the closest to you in the past two weeks.**
**1 2 3 4 5 6 7 8 9 10**
No Hardship — Tremendous Hardship

**9. Rate how often you feel discouraged about your life.**
**1 2 3 4 5 6 7 8 9 10**
Always — Never

**10. Rate your satisfaction with your work and your jobs around the house in the past month.**
**1 2 3 4 5 6 7 8 9 10**
Very Dissatisfied — Very Satisfied

**11. How uncomfortable do you feel today?**
**1 2 3 4 5 6 7 8 9 10**
Not At All — Very Uncomfortable

**12. Rate in your opinion, how disruptive your cancer has been to those closest to you in the past two weeks.**
**1 2 3 4 5 6 7 8 9 10**
Totally Disruptive — No Disruption

**13. How much is pain or discomfort interfering with your daily activities?**
**1 2 3 4 5 6 7 8 9 10**
Not At All — A Great Deal

**14. Rate the degree to which your cancer has imposed a hardship on you (personally) in the past two weeks.**
**1 2 3 4 5 6 7 8 9 10**
Tremendous Hardship — No Hardship

**15. How much of your usual household tasks are you able to complete?**
**1 2 3 4 5 6 7 8 9 10**
All — None

**16. Rate how willing you were to see and spend time with those closest to you in the past two weeks.**
**1 2 3 4 5 6 7 8 9 10**
Unwilling — Very Willing

**17. How much nausea have you had in the past two weeks?**
**1 2 3 4 5 6 7 8 9 10**
None — A Great Deal

**18. Rate the degree to which you are frightened of the future.**
**1 2 3 4 5 6 7 8 9 10**
Constantly Terrified — Not Afraid

**19. Rate how willing you were to see and spend time with friends in the past two weeks.**
**1 2 3 4 5 6 7 8 9 10**
Unwilling — Very Willing

**20. How much of your pain or discomfort over the past two weeks was related to your cancer?**
**1 2 3 4 5 6 7 8 9 10**
None — All

**21. Rate your confidence in your prescribed course of treatment.**
**1 2 3 4 5 6 7 8 9 10**
No Confidence — Very Confident

**22. How well do you appear today?**
**1 2 3 4 5 6 7 8 9 10**
Extremely Poor — Extremely Well

**23. How would you rate your overall quality of life over the past two weeks?**
**1 2 3 4 5 6 7 8 9 10**
Life Couldn't Be Worse — Life Couldn't Be Better

*© 2001 Cancer Research Campaign*
